# Peer Review of “Google Trends as a Predictive Tool for COVID-19 Vaccinations in Italy: Retrospective Infodemiological Analysis”

**DOI:** 10.2196/38726

**Published:** 2022-04-19

**Authors:** Angela Chang

**Affiliations:** 1 Department of Communication University of Macau Macao Macao

**Keywords:** COVID-19, epidemiology, Google Trends, infodemiology, infoveillance, Italy, public health, SARS-CoV-2, vaccinations, vaccines, social media analysis, social media

*This is a peer-review report submitted for the paper “Google Trends as a Predictive Tool for COVID-19 Vaccinations in Italy: Retrospective Infodemiological Analysis.”*

## Round 1 Review

This brief paper [1] examines the effective approach to investigating vaccine adherence against COVID-19 via Google Trends. The topic is interesting and important to provide actionable data to the World Health Organization or other related health organizations to prioritize their risk communication efforts. The manuscript is nicely written and easy to understand. These data are of potential interest, but there are some concerns.

### Major Comment

1. The methodological strength is poor. It should discuss the overarching sampling method, measures, and procedures to justify the Google and news media content in this study.
2. In line with the methodology concern, the chosen keywords are questionable too.
3. Additionally, there is no rationale for sampling the historical archive of the newspaper “La Repubblica.” Is this the second most read Italian newspaper online?
4. Confounding is a statistical concept that is important to all researchers. The concept of confounding is explained with the help of an amusing but true example. The methods to deal with confounding should be more detailed, with more applications and disadvantages to be examined.
5. The role of the mass media was considered as a confounding factor. Actually, confounding is said to exist when a third factor, known as the confounding variable, explains the association between two variables. One of the results indicated that vaccine reservation queries (VRQs) and news about COVID-19 vaccines have been low and characterized by lags. I am afraid this could be a failure to identify and control for confounding, which could result in the faulty interpretation of study outcomes. So, you really can’t say for sure whether the lack of news influence (ie, from one specific website only) leads to the unwillingness of vaccination.
6. Another study outcome linked the VRQs and vaccinated for their positive linear relation. Instead of a valuable research question, it sounds like common sense that most laymen would agree with.
7. Following the abovementioned concern, it is not sustainable that the conclusion shows that Google Trends is a surveillance and prediction tool for vaccine adherence against COVID-19 in Italy.

### Minor Comments

1. Please list the ethics issue for this study if approved.
2. The first letters of a term should correspond to the initials, for example, “vaccine reservation” (VRQ).

## Abbreviations

VRQ: vaccination reservation query
